# Molecular signatures of aggressive pediatric liver cancer

**Published:** 2021

**Authors:** Michael E. Johnston, Nikolai Timchenko

**Affiliations:** 1 Division of General and Thoracic Surgery, Cincinnati Children’s Hospital Medical Center, USA; 2 University of Cincinnati, Ohio, Cincinnati, 33333 Burnet Ave, 45229, USA

## Abstract

Liver masses account for 5 to 6% of pediatric cancer, which includes hepatoblastoma (HBL) along with rare cases of hepatocellular carcinoma (HCC). The most dangerous form of pediatric liver cancer is aggressive HBL, which can be characterized by chemo-resistance and multiple nodules or metastases at diagnosis, all correlating with worse clinical prognosis. Despite intensive studies and a significant improvement in overall outcomes, very little is known about the key molecular pathways which determine the aggressiveness of pediatric liver cancer. Although genetic mutations have been reported in aggressive HBL, they represent a low level (1.9% per case) and are found mainly in two genes CTNNB1 and NRF2. Over the past 5 years, our liver biology and tumor group at Cincinnati Children’s Hospital Medical Center has investigated molecular signatures of aggressive HBL by examination of fresh tissue specimens, which were studied immediately after surgery to preserve the integrity of key biochemical pathways. Summarization of these high quality HBL samples discovered several critical pathways that are specific for aggressive pediatric liver cancer. These pathways include three characteristics:

1. Conversion of tumor suppressor proteins (TSPs) by posttranslational modifications into oncogenes
2. Activation of specific chromosomal regions, i.e., Aggressive Liver Cancer Domains (ALCDs) within many oncogenes, resulting in increased expression of oncogenes
3. Potential epigenetic mechanisms that open chromatin structure of oncogenes via ALCDs. This commentary summarizes our key findings and discusses development of potential ALCD-based therapeutic approaches.

## Aggressive HBL: Clinical Features and Genomic Mutations

Although there are many preclinical investigations of HBL and several clinical trials, aggressive features of HBL have not been summarized to define aggressive *vs.* non-aggressive HBL. In our studies of HBL at CCHMC, we defined aggressive HBL as displaying clinically aggressive features including chemo-resistance, relapsed HBL, multiple nodules at diagnosis, vascular invasion, and metastatic at diagnosis [1]. Our work which has included engraftment of patient derived xenografts (PDXs) of these tumors has aligned with Nicolle et al. who showed that successful engraftment of these tumors correlates with worse clinical prognosis [2]. A recent report by Cairo et al. analyzed 174 HBL patients with the goal to integrate known biomarkers into a tool which can predict survival of HBL patients [3]. In agreement with previous findings [4,5], the authors confirmed that HBL has a low level of genetic mutations mainly in the CTNNB1, NRF2 genes, and TERT genes [3]. The authors also found that 36% of analyzed patients have “a 16-gene signature” previously suggested for prediction of worse outcome [5]. However, they did not find associations of CTNNB1 mutations with clinical or pathological features. Given this information, it is clear that there is a need for discovering additional biochemical alterations which contribute to the development of aggressive HBL. It is also necessary to elucidate how the expression of mutant proteins, such as CTNNB1, NRF2 and TERT1, is increased in aggressive HBL. Our group is working with elucidation of these key intermolecular alterations.

## Aggressive HBL Converts Tumor Suppressor Proteins into Oncogenes

**Figure 1:**
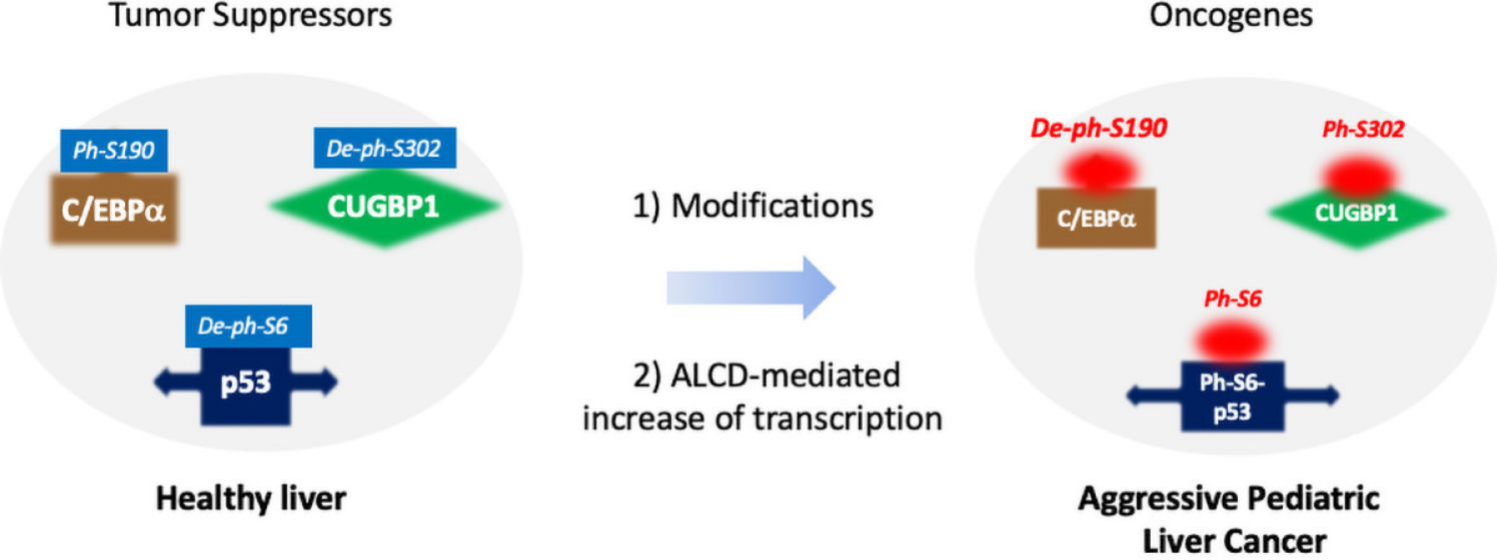
Aggressive HBL converts tumor suppressor proteins C/EBPα, CUGBP1 and p53 into oncoproteins. In healthy livers, major portions of these proteins are phosphorylated or de-phosphorylated at specific Ser residues and work as tumor suppressors. In aggressive HBL, phosphorylation/de-phosphorylation of C/EBPα, CUGBP1 and p53 is changed and the proteins possess oncogenic activities.

The normal quiescent liver is equipped with a set of molecules which support essential liver functions which also aid in protecting the liver from the development of cancer. One group of these molecules are TSPs. Particularly, normal liver expresses high levels of strong inhibitors of proliferation along with TSPs: Rb, p53, C/EBPα, HNF4α and CUGBP1. In a significant amount of our cases of liver cancer, these proteins are downregulated by silencing of promoters and degradation by the ubiquitin proteasome system [1]. In the course of blinded studies of these specimens, we surprisingly found a group of HBL patients who had an elevation of C/EBPα, CUGBP1, HNF4α and p53. Following review of their clinical records, we discovered that this group represents patients with features of chemo-resistance, relapsed HBL, multiple nodules at diagnosis, vascular invasion, or metastases. In our paper [1] and in two other manuscripts, we have examined modifications of these elevated TSPs and their biological activity (Figure 1). Briefly, **C/EBPα:** tumor suppressor C/EBPα is dephosphorylated at Ser190 in aggressive HBL and in some cases of HCC [1,6]. We generated C/EBPα-S193A knock-in mice (mouse Ser193 is equivalent of human Ser190) in which the C/EBPα is inactivated and found that S193A mice developed spontaneous liver cancer through de-differentiation of hepatocytes into cancer-like stem cells [7]. Several reports showed that C/EBPα possesses oncogenic activities under specific settings [6,8]. **CUGBP1:** De-ph-S302-CUGBP1 is a tumor suppressor but is eliminated by Gankyrin in classic HBL [9]. In aggressive HBL, CUGBP1 is phosphorylated at Ser302 and works as oncogene [1,10]. **P53:** Well-known TSP p53 was found to be phosphorylated at Ser6 and elevated in aggressive HBL [1]. Our new observations show that ph-S6-p53 possesses oncogenic activities (Johnston, Hepatology, under review). Thus, these recent studies revealed that one of the molecular characteristics of aggressive liver cancer is the conversion of tumor suppressor proteins into oncoproteins.

## Aggressive Pediatric Liver Cancer Increases Expression of Multiple Oncogenes via Activation (Opening) of Human Genome Regions Called Aggressive Liver Cancer Domains (ALCDs).

**Figure 2:**
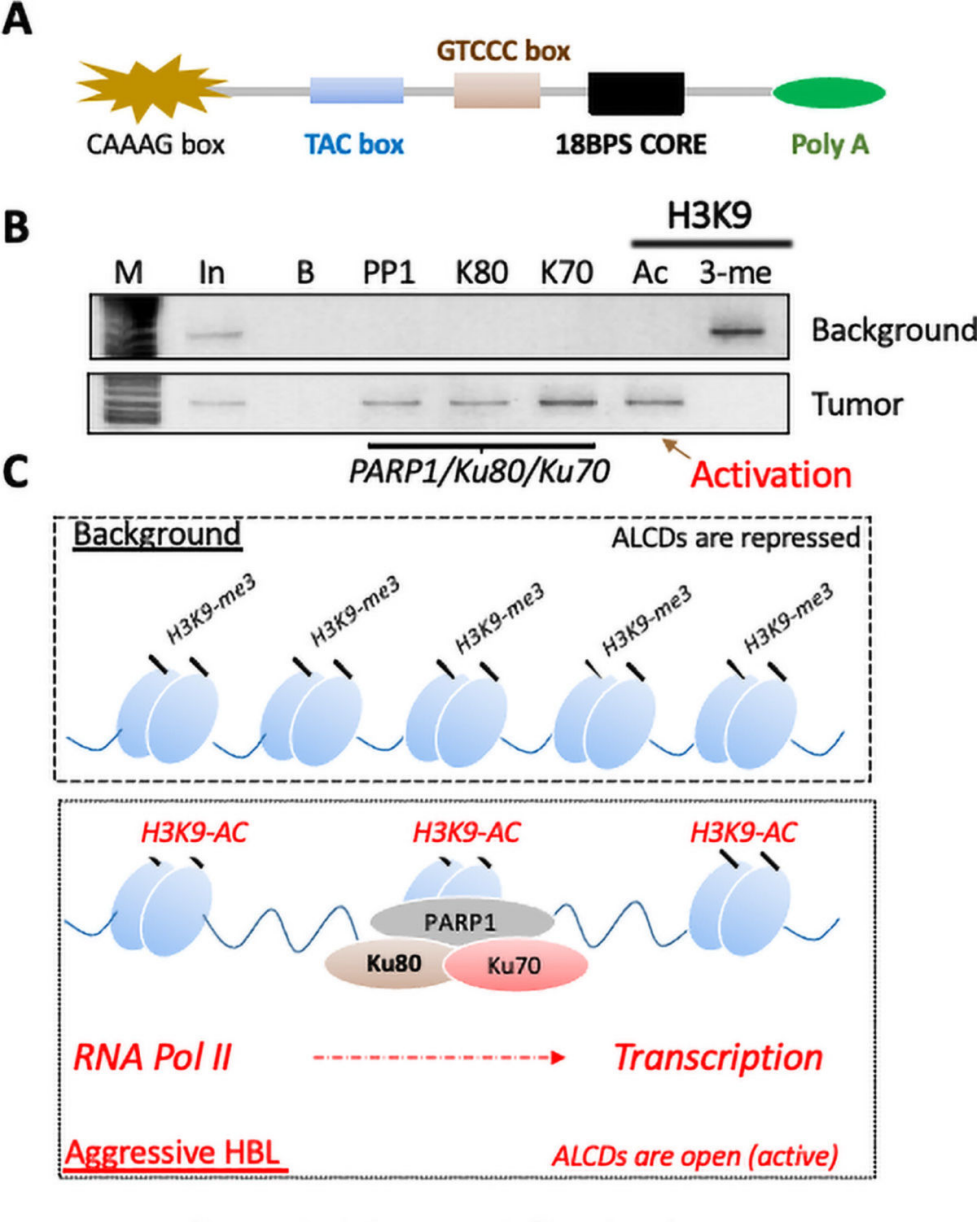
Aggressive Liver Cancer Domains are activated and are open for transcription. A) Structure of ALCDs. 100% homological regions are shown. B) A typical ChIP assay of the ALCDs. Tumor; a tumor section of HBL patient, background; a “healthy” region of liver of the same patient adjacent to tumor, B; beads, PP1; IP with antibodies to PARP1; Ac and 3-me; IPs with antibodies specific to histone H3 acetylated or 3-methylated at K9. C) Summary of the studies which suggest changes of chromatin structures of ALCDs in aggressive HBL.

Our studies of the mechanisms by which aggressive HBL increases oncogenic forms of tumor suppressors found specific chromosomal DNA regions (ALCDs) that are observed only in the human genome. These regions are approximately 250 bp long and are located in introns of genes of many oncoproteins and proteins that accelerate development of liver cancer. ALCDs have 85–95% homology between them and contain short 100% homological regions (boxes) which are shown in Figure 2A. Careful molecular analyses showed that the 18S BP core is occupied by PARP1-Ku80/70 complexes. A typical ChIP assay with ALCD is shown in Figure 2B. The binding of the PARP1 complexes correlates with acetylation of histone H3 at K9, which usually demonstrates open chromatin being transcribed by RNA Pol II (Figure 2C).

### Biological significance:

Identification of ALCDs as human genome regions that enhance liver cancer and promote aggressiveness is highly significant due to a very broad spectrum of genes that might be activated through ALCDs along with the role these oncogenes have in pediatric liver cancer. First, as we mentioned above, ALCDs were first found as regions that increase expression of oncogenic forms of TSPs: C/EBPα, CUGBP1, and p53. In addition, other tumor suppressors such as HNF4α and HACE1 have been found to be modified in aggressive HBL and potentially might work as oncogenes [1]. Second, ALCDs are observed in beta-catenin and NFR2 genes. These two genes are on the top list of genes which are mutated in patients with HBL. The previously described pathways of activation of beta-catenin include mutations within the protein beta-catenin and translocation of the mutant beta-catenin protein from the cell surface to nucleus. In the nucleus, beta-catenin interacts with the transcription factor TCF4 and activates expression of cancer genes. However, it has been shown that wild type beta-catenin can also induce liver cancer if it is overexpressed [11]. Therefore, the increase in transcription of beta-catenin is a key event for oncogenic functions of beta-catenin and mechanisms of its elevation need to be determined. Our finding that the beta-catenin gene contains chromatin ALCDs provides a potential mechanism. NFR2 is also mutated and elevated in patients with poor prognosis and low survival [4,5]. The identification of an ALCD in the NRF2 genes might additionally explain how the mutant form of NRF2 is elevated in aggressive HBL. Among the multiple other ALCD containing genes, many are involved not only in liver cancer, but also in other malignancies. Most importantly, these include Rb, WNT98, GSS, RUNDC1, ANK4 and MYO18B.

## PARP1-Dependent Mechanisms of Activation of ALCD-containing Oncogenes

Our manuscript [1] found that PARP1 is associated with open ALCDs in patients with aggressive HBL. PARP1 is a nuclear protein which has been initially identified as an enzyme involved in repair of double-stranded DNA breaks [12]. However, PARP1 is also a potent transcriptional regulator [12]. This activity is associated with the regulation of transcription factors, change of the chromatin structure, and direct interactions with chromatin remodeling proteins. It is important that PARP1 also interacts with complexes of RNA pol II [13]. The known transcriptional activities of PARP1 which are involved in promotion of cancer include a) interactions with and activation of promoters of key pluripotency genes protecting these genes from epigenetic repression [14]; b) binding to a transcription factor E2F1 and functioning as a strong activator of E2F1 [15]; and c) modulation of chromatin on the c-myc promoter leading to activation of c-myc gene [16]. Given the location of ALCDs mainly in introns, we suggest that these transcriptional activities of PARP1 might be, at least partially, involved in providing long distance interactions between ALCDs and promoters with subsequent activation of promoters. We are currently investigating such mechanisms of activation of oncogenes by ALCDs. Another pathway by which activation of oncogenes containing several ALCDs might be through a simple opening of chromatin throughout entire genes and subsequent facilitation of transcription. In this regard, we have found that the Glypican 3 gene, which is highly elevated in many HBL and HCC patients, contains six ALCDs (unpublished data). We suggest the opening of these ALCDs might be involved in high overexpression of Glypican 3 in patients with aggressive HBL.

Future studies and development of potential ALCD-based therapeutic approaches: Our studies showed that inhibitors of PARP1, Olaparib (Ola), and DPQ, inhibit proliferation in hepatoblastoma HepG2 and Huh6 cells [1]. Moreover, our recent work with PDXs showed that Ola partially inhibits tumor growth with reduction in proliferation in HBL-PDX models (Johnston, Hepatology, under review). Therefore, we have obtained substantial evidence that Ola might be considered for ALCD-based clinical trials with aggressive HBL patients. In this regard, the inhibitor of PARP1, Ola, is in trials for several human cancers including ovarian cancer [17], prostate cancer [18]; and breast cancer [19]. There are also several recent reports showing promising development of Ola-based therapy for liver cancer, including aggressive HBL [20–22]. Positive results in these trials and studies are likely associated with Ola-mediated repression of ALCD-dependent oncogenes. It is also clear that Ola alone is not sufficient to inhibit the HBL completely due to complexity of the regulation of ALCDs. First, PARP1 and other known components of the PARP1 complex Ku70/Ku80 do not bind to DNA in a sequence specific manner and should be delivered to ALCDs by transcription factors (TFs). Therefore, identification of these factors is required. Second, ALCDs contain several additional 100% homological (20–25 nucleotide) regions (see Figure 2) which might be bound by other TFs and activated by other signaling pathways. It is likely that these unknown TFs and pathways are also involved in activation of ALCD-containing oncogenes and must be dampened or eliminated for successful inhibition of ALCD-dependent cancers including aggressive HBL. As a final remark, we would like to emphasize that ALCDs have only been observed in the human genome. This feature creates challenges in studies of aggressive HBL using genetically modified mouse models and strongly suggests that the inhibition of ALCD-dependent liver cancer can be examined only in human cell culture studies or PDXs derived from human cancers.
